# Therapeutic Applications of Curcumin in Diabetes: A Review and Perspective

**DOI:** 10.1155/2022/1375892

**Published:** 2022-02-02

**Authors:** Cristina Quispe, Jesús Herrera-Bravo, Zeeshan Javed, Khushbukhat Khan, Shahid Raza, Zehra Gulsunoglu-Konuskan, Sevgi Durna Daştan, Oksana Sytar, Miquel Martorell, Javad Sharifi-Rad, Daniela Calina

**Affiliations:** ^1^Facultad de Ciencias de la Salud, Universidad Arturo Prat, Avda Arturo Prat 2120, Iquique 1110939, Chile; ^2^Departamento de Ciencias Básicas, Facultad de Ciencias, Universidad Santo Tomas, Chile; ^3^Center of Molecular Biology and Pharmacogenetics, Scientific and Technological Bioresource Nucleus, Universidad de La Frontera, Temuco 4811230, Chile; ^4^Office for Research Innovation and Commercialization, Lahore Garrison University, Lahore, Pakistan; ^5^Atta-ur-Rahman School of Applied Biosciences, National University of Sciences and Technology, Islamabad, Punjab, Pakistan; ^6^Faculty of Health Science, Nutrition and Dietetics Department, Istanbul Aydin University, Istanbul 34295, Turkey; ^7^Department of Biology, Faculty of Science, Sivas Cumhuriyet University, 58140 Sivas, Turkey; ^8^Beekeeping Development Application and Research Center, Sivas Cumhuriyet University, 58140 Sivas, Turkey; ^9^Department of Plant Physiology, Slovak University of Agriculture, A. Hlinku 2, 94976 Nitra, Slovakia; ^10^Department of Plant Biology, Institute of Biology, Kiev National, University of Taras Shevchenko, Volodymyrska, 64, 01033 Kyiv, Ukraine; ^11^Department of Nutrition and Dietetics, Faculty of Pharmacy, and Centre for Healthy Living, University of Concepción, 4070386 Concepción, Chile; ^12^Facultad de Medicina, Universidad del Azuay, Cuenca, Ecuador; ^13^Department of Clinical Pharmacy, University of Medicine and Pharmacy of Craiova, 200349 Craiova, Romania

## Abstract

Diabetes is a metabolic disease with multifactorial causes which requires lifelong drug therapy as well as lifestyle changes. There is now growing scientific evidence to support the effectiveness of the use of herbal supplements in the prevention and control of diabetes. Curcumin is one of the most studied bioactive components of traditional medicine, but its physicochemical characteristics are represented by low solubility, poor absorption, and low efficacy. Nanotechnology-based pharmaceutical formulations can help overcome the problems of reduced bioavailability of curcumin and increase its antidiabetic effects. The objectives of this review were to review the effects of nanocurcumin on DM and to search for databases such as PubMed/MEDLINE and ScienceDirect. The results showed that the antidiabetic activity of nanocurcumin is due to complex pharmacological mechanisms by reducing the characteristic hyperglycemia of DM. In light of these results, nanocurcumin may be considered as potential agent in the pharmacotherapeutic management of patients with diabetes.

## 1. Introduction

Diabetes is an indicator present in humans with elevated levels of blood glucose. These humans are not able to metabolize glucose due to defects in insulin secretion and/or action [1, 2]. Not only environmental and genetic factors are responsible for diabetes, but also many other factors including insufficient physical activity, overconsumption of food and drinks, obesity, stress, and industrialization can affect the development of diabetes [3]. The chronic hyperglycemia can cause other complications with long-term damage, dysfunction, and organ damage especially the eyes, kidneys, nerves, heart, and blood vessels [4, 5].

Diabetes can be categorized as type 1 diabetes (T1D), type 2 diabetes (T2D), gestational diabetes, and other specific types of diabetes. T1D and T2D are two major etiopathogenetic categories of diabetes [6, 7]. T1D is caused by an absolute deficiency of insulin secretion. T2D, a much more prevalent category, is caused by a combination of resistance to insulin action and insufficient compensatory insulin secretory response [8]. With weight loss, exercise, and/or oral glucose-lowering agents, sufficient glycemic control can be achieved in diabetics. However, individuals with no residual insulin secretion due to extensive *β*-cell destruction require insulin to survive [4]. *β*-Cells produce insulin to control the blood glucose level which can be effectively controlled by restoring and replacing the *β*-cell mass. In diabetes, the complications can be spread to the body by oxidative stress in the diseased person [9, 10]. One of the reasons for decreasing insulin secretion by pancreatic *β*-cells is increasing free radicals and oxidative stress in the human body [11]. Therefore, antioxidant therapy may play an important role in diabetes therapy [12]. Studies have shown that curcumin can play a critical role in the cure of diabetes by attempting to prevent the decrease of *β*-cell functions due to its high therapeutic properties [13].

Curcumin (diferuloylmethane chemical structure) is the major curcuminoid found in the turmeric root. Curcuma plant (*Curcuma longa* L.) is a perennial herb of the Zingiberaceae family [14]. The tuberous rhizome of turmeric is used as a spice (capable of replacing ginger), dye, and medicinal plant [15]. It is one of the main ingredients in Indian curry. The native land of turmeric, probably India, is not found in the wild anywhere else. It was around 500 BCE that turmeric appeared as a significant part of Ayurvedic medicine.

**Figure 1:**
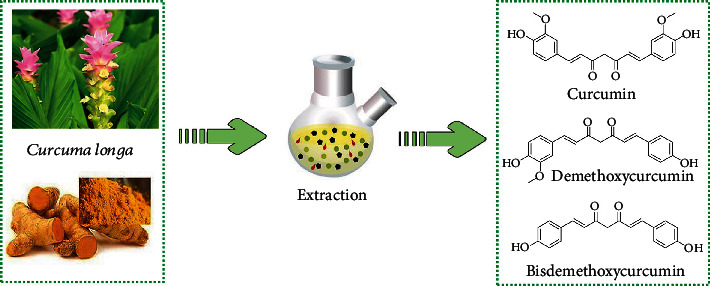
Chemical structures of most important curcuminoids.

It is known that the three important curcuminoids are curcumin, demethoxycurcumin, and bisdemethoxycurcumin (Figure 1) [16].

Of these, curcumin is the most active and most significant to health [17]. It is important to admit the difference between turmeric and curcumin solutions or extracts. Turmeric contains curcuminoids, which are bioactive compounds, and curcumin is one of these curcuminoid compounds [17]. While turmeric contains only 2–9% curcuminoids, 75% of these active curcuminoids are curcumin [17, 18].

The curcumin molecule exhibits a multitargeting ability in various pathological conditions, which allows translation into a therapeutic or nutraceutical agent. Curcumin can support balance for oxidative and inflammatory conditions, metabolic syndrome, arthritis, anxiety, and hyperlipidemia [19]. Curcumin has antioxidant, antiamyloid, antimicrobial, antineoplastic, immune-modulating, and neuroprotective effects [20, 21]. Curcumin also showed antidepressant activity through modulating the release of serotonin and dopamine [22].

The therapeutic curcumin limitations have been well known for a long time, low solubility, low stability, poor bioavailability, low penetration, rapid metabolization, and targeting efficacy.

In recent times, nanomedicine has appeared as a therapeutic option because of its ability to encapsulate nanoformulations and their delivery to target tissue sites in a precise manner [23, 24]. Furthermore, nanomedicines have limited cytotoxicity and high specificity which makes them an excellent choice for the prevention and remediation of various biological diseases. Therefore, nanotechnology offers new opportunities to solve these problems, and new curcumin nanoparticles and liposomes have been designed.

In this present article, we aim to review and underline different studies and approaches looking into the promising role of nanocurcumin in diabetes treatment with emphasis on their formulation characteristics, general bioactivity, and experimental evidence and describe the questions and opportunities in developing these systems.

## 2. Review Methodology

For this review, databases such as PubMed/MEDLINE and ScienceDirect were accessed. The following MeSH terms were used to search: “Curcumin/therapeutic use,” “Diabetes Mellitus,” “Experimental/pathology,” “Diabetes Mellitus,” “Experimental/complications,” “Nanoparticle/chemistry,” “Drug Carriers/chemistry,” “Wound Healing/drug effects,” “Animals,” and “Humans.” In the last decade, up to 23905 papers until 2021 (source: ScienceDirect) that have been published in connection with curcumin (2010–2021) in the field of medicinal chemistry, food chemistry, pharmacology, toxicology, biology, biochemistry, bioorganic, and analytical chemistry also appeared [25]. It was two times more than for the previous 10-year period, which says about significant interest in this secondary metabolite.

The taxonomy of the plants was verified according to the PlantList [25, 26].

### 2.1. Inclusion Criteria

The inclusion criteria are as follows: preclinical studies evaluating nanocurcumin in DM, *in vitro* or *in vivo* pharmacological experimental research, studies indicating nanocurcumin concentrations/doses and administration route, and studies describing the molecular mechanism(s) of nanocurcumin in DM. Studies that met the inclusion requirements were retrieved based on the following criteria: first author's surname, year of publication, kind and technique of study, doses or concentrations examined, route of administration, molecular mechanism, and key findings.

### 2.2. Exclusion Criteria

The exclusion criteria are as follows: duplicate articles; absence of descriptors in title and abstract; and articles outside the main topic, abstracts, letter to editors, and papers which included homeopathic agents.

## 3. Extraction Methods of Curcumin

The structural characteristics and features of curcumin can be affected by the extraction method. Conventional (Soxhlet extraction, hydrodistillation, and maceration) and novel techniques (ultrasound-assisted extraction, microwave-assisted extraction, high hydrostatic pressure extraction, supercritical fluid extraction, enzyme-assisted extraction, zone refining, and dipping methods) can be used for curcumin extraction from turmeric roots [27].

The conventional extraction methods have some limitations such as applying high temperature which is not beneficial for thermosensitive compounds, using a large volume of organic solvent, long time, and low extraction yield [28]. Due to these drawbacks, researchers tend to use other techniques which could incorporate high extraction efficiency and environmentally friend technology [29].

Microwave-assisted extraction, ultrasound-assisted extraction, supercritical fluid extraction (requiring expensive instruments), pressurized liquid extraction, and enzyme-assisted extraction have been reported as common advanced methods for curcumin extraction [17, 27].

Recent studies about the extraction of curcumin by novel techniques have shown that novel techniques have better extraction yield, shorter time, and higher antioxidant activity. Choi et al. [30] showed that high hydrostatic pressure is a promising method to obtain higher antioxidant activity from turmeric, and also, they observed higher concentration of vanillic and ferulic acid content in extracts with increasing time. A comparative study conducted by Wakte et al. [31] reported that microwave-assisted extraction is a more efficient method for extraction of curcumin from *C. longa* among Soxhlet, ultrasonic, and supercritical carbon dioxide-assisted extractions in terms of yield and required time.

In the same study, the extraction yield was reported with the decreasing order as follows: microwave-assisted extraction (90.47%), ultrasound-assisted extraction (71.42%), supercritical carbon dioxide extraction (69.36%), and Soxhlet extraction (2.1%). Additionally, Liang et al. [32] have also proved that ionic liquid-based microwave-assisted extraction is a fast, effective, and pollution-free method for curcumin extraction.

## 4. Bioavailability of Curcumin

Bioavailability is defined as the part (percentage) of a given dose of the drug that reaches the circulatory system (systemic circulation) [33].

In all cases of use of a medicinal product, it is necessary that the active substance of the medicinal product, known as the “pharmaceutically active compound” (CFA), be able to reach the body. In order to have a therapeutic effect, however, it is not enough for the active substance to enter the body. The active substance must be available in the correct dose in the area of the body in which it is to act. This area of the body is called the “target area” [33]. The active substance must also reach the target area within a certain time frame and remain there for a certain period. In the case of intravenous injection, when the medicine is administered directly into the circulatory system, the bioavailability is considered to be 100% [34].

Curcumin is characterized by poor solubility and poor absorption in the free form in the gastrointestinal tract, and its rapid biotransformation to inactive metabolites greatly limit its utility as a health-promoting agent and dietary supplement. In recent years, several nanoformulation-based methods have been conducted for improving curcumin use *in vitro* and *in vivo* studies involving the use of adjuvants, stabilizers, conjugates/polymer conjugates, lipid/liposomes, hydro-/micro-/nanogels, and nanoparticles [35]. Recent experimental studies in curcumin nano- and microformulations with highly increased absorption showed a desirable blood level of the active forms of curcumin [36].

The nanoformulations of curcumin may be possible to use for a wide range of potential applications, including prophylaxis of diabetes, pain management, and protection of tissue [36, 37]. The curcumin formulation is comprised of liquid droplet nanomicelles containing Gelucire®, and polysorbate 20 (BioCurc®) has been shown to have the highest bioavailability with an absorption >400-fold as compared to unformulated curcumin [38]. Therefore, nanotechnology can help to overcome curcumin efficiency problems like solubility, toxicity, rapid drug metabolism, degradation, and drug stability [35].

## 5. Nanotechnological Approaches to Improve Curcumin Bioavailability

Nanomedicine and delivery systems can be defined as means for transmission of the nanoscale therapeutic agents to a target site in a specific and precise manner [39, 40]. It is a rapidly emerging science where nanoscale materials can serve both as diagnostic and therapeutic tools [41, 42].

Nanotechnology is a promising field with vast benefits ranging from precise medicines to advance polymers designed for strengthening fibres and components for heavy-duty machinery [15, 43]. Nanomedicines are site-specific, precise, and target-oriented with limited cytotoxicity which, therefore, can be employed to treat chronic human diseases. This has led to the use of these nanoscale materials in the development of chemotherapeutics, immunotherapeutic agents, and biological agents for treating various diseases [44, 45].

Advances in nanotechnology have reduced the gap between biological and physical sciences through the application of nanostructures such as nanomedicine and nanodrug delivery platforms [46]. Nanomaterials are small in size ranging between 1 and 100 nm and have revolutionized the field of nanomedicine [47].

These nanostructures are employed in the development of biosensors, microfluidic systems, drug delivery, tissue engineering, and microarray testing [47]. Since nanoformulations are constructed at atomic and molecular levels, they are usually termed nanospheres and are easily absorbed in cellular levels. Nanospheres have unique structural, chemical, mechanical, magnetic, electrical, and biological properties [48, 49].

### 5.1. Curcumin Nanoformulation Types

For drug delivery, pharmaceutical industries employ various nanoformulations such as liposomes, dendrimers, nanoemulsions, and micelles.

#### 5.1.1. Liposomes

Liposomes are a drug delivery system since 1960 [50]. They have broad applications ranging from pharmaceuticals to cosmetics and are involved in the transportation of a broad range of nanomaterials. They are usually spherical and composed of phospholipids and steroids with sizes ranging between 50 and 450 nm. Liposome vesicles are an excellent source for drug delivery as they have similar compositions of the cell membrane and are biodegradable. Liposomes have been approved by FDA for drug delivery; however, there are still some obstacles regarding the liposome-based drug delivery system that is making their use as drug delivery vehicles expensive and cumbersome [51]. Liposomes are generally divided into conventional liposomes, PEGylated liposomes, ligand-targeted liposomes, and theranostic liposomes [52]. Liposomal curcumin has high stability and targeting properties. Ikiki [53] reported that curcumin-loaded liposomes and lipid-based nanoparticles have successfully been prepared using 1,2-dimyristoyl-sn-glycero-3-phosphocholine and an anionic amphiphile, L-glutamic acid, and N-(3 carboxy-1-oxo propyl)-,1,5-di hexadecyl ester. Bulboaca et al. [54] evaluated liposomal curcumin containing polyethylene glycol.

#### 5.1.2. Polymeric Micelles

Polymeric micelles have gained attention for their ability to slow down drug release in cancer patients. These nanoformulations are usually less than 100 nm in size and are composed of amphiphilic block copolymers that form a core-shell structure. The drug of choice is usually loaded in the hydrophobic core where it is released slowly, thus enabling it to reach the target site [55]. Moreover, these nanoformulations have a narrow distribution that aids them with slow renal excretion and accumulation in the tumour tissue via enhanced permeability and retention (EPR) effect.

Polymeric micelles consist of a core-shell formation in an aqueous solution. The core is usually hydrophobic and can be loaded with desired drugs such as camptothecin, docetaxel, and paclitaxel. The shell which is hydrophilic promotes the solubility of these nanoformulations in water. Polymeric micelles are 100 nm in size with a compact structure that delays their release from the body and make them more efficient in targeting tumour cells via the EPR effect. This core-shell formation of polymeric micelles aids them to avoid interaction with nonspecific biological components [56]. Polymeric micelles are the potent and efficient source for hydrophobic drugs. The compact arrangement and core-coat structure enable these nanoformulations to enhance the stability of the drugs. This tight conformation also supports the bioavailability of the drug. Two methods are employed extensively for the synthesis of polymeric micelles. The most convenient approach involves the solvent-based direct dissolution of polymer proceeded by dialysis for extraction. The second method involves precipitation that involves the addition of solvent to a block [57].

#### 5.1.3. Dendrimers

Dendrimers are nanoformulations that have high bifurcation properties, are monodispersible, and are well-defined in their structure. They have a globular shape and can be modulated according to the drug of choice. These properties make dendrimers an excellent choice for drug delivery [58]. Dendrimers are classified into various categories. Based on their function, the dendrimers can be divided into glycodendrimers, peptides, core-shell dendrimers, chirals, liquid crystals, PPI, and PAMAM moieties.

For oral delivery of dendrimer-based nanoformulation, the PAMAM moieties have been studied extensively. PAMAM are hydrophilic which therefore easily travel across the epithelial cells [59]. The drug can be delivered by the dendrimers either via breaking the covalent linkages between the drug and dendrimer by suitable enzymes or via alteration in the physical environment such as the pH and temperature [60]. Dendrimers due to their efficient carrying capacity and rapid drug delivery have been adopted as a model for drug delivery to the tissues of the skin, oral cavity, lungs, and eyes. In addition to this, metallic nanoparticles and quantum dots have also emerged as a potent source for drug transmission as well as imaging in nanomedicine [61].

#### 5.1.4. Nanoemulsions

Nanoemulsions are the most used nanoformulations in pharmaceutical industries [62]. These nanoformulation droplets have a size between 20 and 200 nm and are relatively stable compared to other nano-based formulations. In addition to this, nanoemulsion possesses optical transparency and shelf stability which is necessary for extending drug performance [63].

#### 5.1.5. Nanosuspensions

Nanosuspensions are a promising method that plays an essential role in drug delivery systems to enhance the dissolution of poorly water-soluble compounds. These nanosuspensions hold the pharmaceutically active ingredient stable with the added stabilizers at submicron levels in a liquid phase. Nanosuspensions possess a range of desirable characteristics that permits efficient drug solubility, increased absorption, and adherence to intestinal walls [64].

Nanosuspensions can be prepared by different techniques including precipitation methods, milling methods, and homogenization methods. Preparation of nanosuspensions has been reported to be cost-effective and simple and also to help obtain physically stable products [65]. As a result of rapid solubility and high penetration through the cell wall, nanosuspensions of curcumin prepared with homogenization technique have great potential for a protective and therapeutic role in diabetic cardiomyopathy and other cardiovascular disorders [66].

#### 5.1.6. Nanogels

Curcumin nanogels have attracted a lot of attention due to their high loading capacity, biocompatibility, and responsive release property [67]. Curcumin-loaded collagen-HPMC nanogels have shown great potential and good stability in wound healing applications [68]. Another study related to curcumin-loaded gelatin hydrogels indicated that with the help of nanotechnology, both antioxidant effect and the migration promoting the ability of curcumin can be substantially increased [69].

#### 5.1.7. Nanocrystals

Nanocrystals are solid with sizes ranging up to 1000 nm. They are solely composed of drugs without any carrier molecules [70]. Nanocrystal formulations are stabilized by the surfactants. In addition to this, metallic nanoparticles and quantum dots have also emerged as a potent source for drug transmission as well as imaging in nanomedicine [71].

### 5.2. Polymer-Based Drug Delivery Systems

#### 5.2.1. Synthetic Polymers

Synthetic polymers are being investigated as nanoparticle-forming materials. Commonly used polymer among them is synthetic polylactide-co-glycolide (PLGA) ([72–74]. It is possible to change molecular weight, lactic acid/glycolic acid ratio, and end group of PLGA to alter the physicochemical property, biodegradation rate, and in vivo behaviour [75]. Experimental studies have shown that PLGA-loaded curcumin nanoparticles have higher bioavailability and therapeutic efficacy of the antioxidant molecules [76, 77]. In a recent study, nanosuspensions of curcumin prepared in polylactide-co-glycolide (PLGA) showed an increase in oral bioavailability, reduction in toxicity, and decrease in enzymic and nonenzymic degradation resulting from lower blood glucose level in STZ-induced rats compared to control groups which fed directly with curcumin [74].

#### 5.2.2. Natural Biopolymers

*(1) Chitosan*. Chitosan is a biopolymer extensively used as a continued drug release system due to its ability to adhere to mucosa [78]. The mucoadhesive property along with its ability to slow release of the drug makes chitosan an efficient nanomaterial for drug delivery. Chitosan containing nanomaterials have been extensive as a drug delivery system for various epithelial-related disorders. This drug delivery system has been adopted for prolonged drug release in the epithelial cells of the oral cavity [79], nasal cavity [80], ocular cavity [81], and tracheal epithelial cells [82].

It has been reported that chitosan/sodium tripolyphosphate and hyaluronic acid-based nanoformulation dissolved in an isotonic solution of HPMC (0.75% w/w) successfully delivered ceftazidime to the target site [83]. In addition to this, it was also observed that chitosan-based nanoformulation had the least viscosity when it contacted mucin. Furthermore, chitosan-based nanoparticle carriers increased drug retention capacity, slowed down drug release, and prevented bacterial growth in ocular mucosa. Chitosan-based nanoformulations had limited cytotoxic effects on ocular mucosa [83]. Therefore, chitosan could be implemented as a useful drug delivery system for ocular diseases.

Another study demonstrated that chitosan nanoformulations had limited cytotoxicity when administrated to oral cell lines as compared to lignite and pectin-based nanoparticles.

The compatibility of each nanoformulation was measured by the solubility of each in a salivary environment. Although chitosan nanoformulations were unstable in the salivary environment as compared to pectin and alginate, yet they showed the least cytotoxicity, and the stability was increased by the addition of zinc ion (II) [84]. Altogether, this study shed light on the use of chitosan-based nanoformulations that could be used for oral transmission of desired drugs but still required plenty of refinements. Chitosan-based nanoformulation such as the carboxymethyl chitosan was able to deliver carbamazepine, an intranasal drug across the blood-brain barrier; thus increasing the drug efficacy and systematic drug exposure. Moreover, carboxymethyl chitosan presented greater encapsulation efficiency and greater retention of drugs in the brain [85].

Another study has revealed that hyaluronic acid-coated chitosan nanoparticles when orally administrated prevented degradation of 5-fluorouracil by stomach acid and transmitted it to the colon with minimal cytotoxicity. This study confirmed that chitosan-based nanoformulations could be used as a drug delivery platform for colon cancer due to their limited cytotoxic effects and a treatment option for colon cancer [86].

It has been reported less lately that positively charged chitosan nanoparticles prepared through the ionic gelatin method and loaded with insulin when administrated subcutaneously showed glycemic regulation in streptozocin-induced diabetic rats up to 98 h. These nanoformulations permitted the release of insulin in acidic pH caused by glucose oxidase and catalase activity both *in vitro* and *in vivo*. These nanoformulations were developed in an injectable form for closed-loop insulin delivery and released insulin in response to elevated levels of glucose [87].

Song et al. [88] prepared an oral insulin formulation composed of chitosan termed insulin-loaded carboxymethyl-beta-cyclodextrin-grafted chitosan. Using Caco-2 cell lines, they established the fact that this newly formed nanoformulation was stable and efficiently released drug in simulated colon environment *in vitro*. Furthermore, they also showed that insulin-loaded carboxymethyl-beta-cyclodextrin-grafted chitosan efficiently reduced sugar levels in diabetic rats [88].

*(2) Alginate*. Alginate is another biopolymer that has been extensively used for drug transmission and drug delivery [89]. Alginates are mucoadhesive with anionic characteristics which makes them strong adherent polymers. Nanoformulation consisting of alginate, insulin, and nicotinamide as releasing agents ameliorated the serum levels of insulin in the diabetic rat when administrated sublingually [86]. This finding indicated that alginate could be used as a potent carrier for drug delivery and excellent pharmacological efficacy.

Alginate nanoformulation when administrated through the intranasal route released venlafaxine at higher blood-brain ratios. Furthermore, venlafaxine absorption was increased several folds when administrated intranasally as compared to the intravenous route. Therefore, alginate could be used as a treatment option for depression [90]. In addition to this, alginate nanocarrier containing epidermal growth factor coated on their surface proved efficient in treating non-small-cell lung carcinoma.

Alginate nanoformulation carrying cisplatin reduced cellular growth faster than the free drug in lung cancer cell lines [91]. Polyelectrolyte complexes (PEC) in combination with alginate nanocomposites were able to deliver insulin in a pH-dependent manner in Caco-2 cell lines. Furthermore, oral administration of PEC/alginate-coated insulin promoted upregulation of insulin in diabetic rats; therefore, PEC alginate combination could be used as a therapeutic option for the treatment of diabetes [92].

*(3) Xanthan Gum*. Xanthan gum is a heteropolysaccharide with excellent adhesive properties. It is a high molecular weight polysaccharide obtained from Xanthomonas campestris with limited toxicity. It is extensively used as an excipient in pharmaceutical industries [93]. Xanthan gum thiolation with L-cystine has been proven effective mucal adhesive for the treatment of sialorrhea. It prolonged drug release and retention in mucoid cells of the buccal cavity compared to normal xanthan gum [94].

Xanthan gum-based thiolates and nonformulation have been reported to modulate angiogenesis and abdominal wall reconstruction. Injectable hydrogels that were composed of xanthan gum and chitosan nanoformulations successfully carried vascular endothelial growth factors to damaged intestinal walls and aided in the process of regeneration [95].

In addition to this, xanthan gum-based nanoformulations aided in the development of excipients for nasal delivery of drugs. Xanthan gum-based thiolate showed greater affinity to the binders such as the nicotinic acid and promoted cellular adhesion to a greater extent as compared to normal xanthan gum [96].

*(4) Cellulose and Hydroxypropyl Methylcellulose*. Cellulose has been extensively used as a drug delivery system because of its varied characteristics, such as the increase in the solubility, chelation, and gelation of drugs. Altogether, these properties permit the slow release of drugs and maximum absorption [97]. Cellulose nanocrystals in combination with the chitosan nanoparticles significantly increased the repaglinide when administrated orally. This combination of cellulose nanocrystals and chitosan facilitated the slow release of the repaglinide as cellulose nanocrystals provided the hydrogen bonding to the drug. This efficacy was greatly increased due to the presence of chitosan as the native cellulose nanocrystals had limited control over the release of the drug [98].

One of the most beneficial types of cellulose is hydroxypropyl methylcellulose (HPMC) which has been widely used in oral controlled drug delivery systems due to its unique properties like low toxicity, extensive contact with the mucosa, highly swellable nature, and ocular wound healing accelerating properties. A recent study showed that the combination of alginate beads with carboxymethyl cellulose loaded with 5-flouroacyl resulted in an increased retention rate of the drug up to 90% in Caco-2 cell lines. Furthermore, carboxymethyl cellulose greatly increased mucoadhesiveness in the simulated colonic environment and increased the release of 5-flouracyl [99].

A series of cellulose-derived nanoformulations (hydroxy methylcellulose, methylcellulose, sodium carboxymethyl cellulose, and hydroxyethyl cellulose) were used to test the delivery of acyclovir in the nasal mucosa. This study confirmed that a combination of either two derivatives in combination with the graft copolymer was useful for the retention of acyclovir in the nasal mucosa as well as its adherence. The drug release time was extended in the presence of hydroxyethyl cellulose [100].

### 5.3. Stabilizers

The stabilizers most commonly employed in the nanoprecipitation method for the formulation of nanoparticles with curcumin are Pluronics [101], polyethylene glycol [54], polyvinyl alcohol [77], cetyltrimethylammonium bromide [73], sodium lauryl sulfate, celluloses, and vitamin E tocopherol polyethylene glycol succinate (vitamin E TPGS) [102].

Pluronics have been found to have a lot of pharmaceutical uses as emulsifiers, surfactants, solubilizing agents, and wetting agents. The physicochemical properties of Pluronics change with the difference in the numbers of polyethylene oxide and polypropylene oxide blocks [101].

Vit E TPGS (D-*α*-tocopheryl polyethylene glycol succinate) has demonstrated enhanced bioavailability of poorly absorbed drugs, vitamins, and micronutrients by acting as an absorption and permeability enhancer. Physical stability is an important criterion for the selection of stabilizers. However, it should be considered that stabilizers are not inactive and they can affect the bioavailability due to the interactions with cells and cell layers [102].

## 6. Benefits of Curcumin and Nanocurcumin in Diabetes: Underlying Mechanisms

In recent years, lots of studies proved that delivering curcumin in the form of nanoparticles and liposomes could help to achieve therapeutic outcomes [35]. Curcumin liposomes can pass the blood barrier, and it can show their potential effect on vascular complications of diabetes. Nanoencapsulation of curcumin is also an efficient way to reduce inflammation in streptozotocin- (STZ-) induced rats [54]. Devadasu et al. [76] achieved a 9-fold increase in bioavailability of curcumin when it nanoencapsulated. In the same study, they reported a decrease in plasma triglycerides, total cholesterol, C-reactive protein (CRP), tumour necrosis factor (TNF)-*α*, and interleukin (IL)-6 levels and an increase in the high-density lipoprotein cholesterol level on diabetic rats given polylactic-co-glycolic acid (PLGA–curcumin nanoparticles for four weeks.

Curcumin improves the pathological events in diabetes through different mechanisms such as regulation of lipid metabolism, antioxidative activity, and antihyperglycemic, anti-inflammatory, and antiapoptotic effects [103]. Studies related to curcumin have shown that curcumin not only mitigates the complications directly caused by diabetes but also alleviates indirect complications like nephropathy, retinopathy, neuropathy, atherosclerosis, stroke, and coronary artery diseases. The common complications of long-lasting diabetes can be ameliorated by the consumption of curcumin [35, 104]. In animal models, curcumin extract delays diabetes development, improves *β*-cell functions, prevents *β*-cell death, and decreases insulin resistance [37]. Some doses of nanocurcumin can be more effective in reducing lipid profile than poor curcumin extract [105].

Chuengsamarn et al. [106] conducted three groups of trials (double-blinded, randomized, and placebo-controlled) which included 240 prediabetic subjects. A capsule containing 250 mg curcuminoids was received by the subjects for 9 months. After the treatment, they reported that 16.4% of subjects in the placebo groups were diagnosed with T2D, while subjects in the group treated with curcumin were not diagnosed with diabetes. An improvement was observed in the overall function of *β*-cells with higher HOMA-*β* (homeostasis model assessment) and lower CRP (C reactive protein). Also, the group treated with curcumin showed an increase in adiponectin levels and a decrease in insulin resistance.

**Figure 2:**
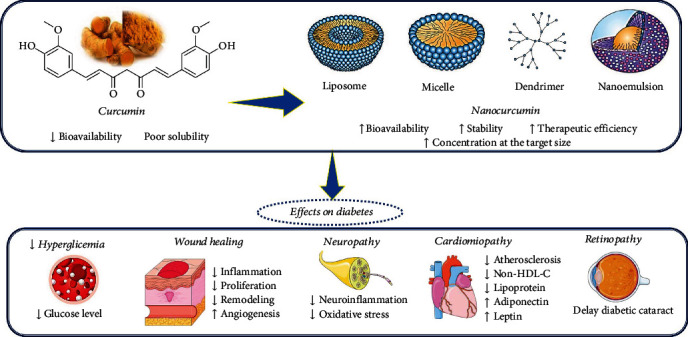
Schematic diagram with the effects of curcumin nanoformulations in diabetes. Abbreviation and symbols: ↑: increase; ↓: decrease; non-HDL-C: non-HDL cholesterol.

Recent studies related to the nanoformulations of curcumin to alleviate symptoms of diabetes and diabetic complications are shown in Figure 2 and Table 1.

### 6.1. Effects on Hyperglycemia

Wickenberg et al. [110] showed that ingestion of 6 g turmeric increased postprandial serum insulin concentrations without significantly affecting plasma glucose levels.

The antihyperglycemic and antihyperlipidemic effects of curcumin-supplemented yoghurt (30-90 mg/kg body weight) have been shown in STZ-induced diabetic rats for 31 days [111]. A study performed by Gutierres et al. [112] showed that curcumin and reduced insulin dose decreased glycemia, dyslipidemia, and biomarkers of liver and kidney damage and improved the activity of hepatic antioxidants (superoxide dismutase and glutathione peroxidase) in STZ-induced rats.

### 6.2. Diabetic Wound Healing

Many people who have diabetes develop wound healing problems [113]. There are usually four stages for the progression of wound healing including hemostasis, inflammation, proliferation, and remodelling [28]. But there is also the possibility of not following these steps leading to inefficient wound recovery [107]. Due to its destructive effects, oxidative stress plays a crucial role in delaying the wound healing process. Curcumin has great wound healing properties by the action of reduced oxidative stress by scavenging the free radicals [69]. Haryuna et al. [114] investigated the antioxidant activity of curcumin on cochlear fibroblasts in STZ-induced rats. Rats were fed with a 0.5% curcumin diet for 3-8 days. They found that dietary curcumin could effectively control oxidative stress by increasing the expression of the superoxide dismutase enzyme.

Akbar et al. [13] developed a formulation that is mixed with polymeric micelles containing curcumin as a wound healing agent. The newly developed curcumin-based formulations have proved excellent healing efficacy. A study conducted by Liu et al. [69] improved a system that contains encapsulated curcumin nanoparticles for accelerating skin wound healing efficiently and safely for diabetics. As a result of the study, both the antioxidant effect and the migration promoting the ability of curcumin significantly increased with the help of nanotechnology. Furthermore, Li et al. [107] indicated that chitosan nanoparticles loaded with curcumin may effectively attenuate inflammation mediated by macrophages and enhance angiogenesis *in vitro* and *in vivo* and further accelerate wound healing in diabetic rats.

### 6.3. Effects on Diabetic Neuropathy

Neuropathic pain is a complication of diabetes that affects diabetic patients. Allodynia and hyperalgesia can also be seen in patients with diabetic neuropathy. Inflammation is one of the main reasons underlying various deficits seen in neuropathy [115–117].

Curcumin may be considered for the treatment of diabetic neuropathic pains due to its therapeutic potential. A study performed by Banafshe et al. [115] reported that curcumin can be used effectively on diabetic peripheral neuropathic pain and possible involvement of the opioid system. In STZ-induced rats, chronic, but not acute, treatment with curcumin prevents weight loss and alleviates mechanical allodynia.

In an *in vivo* study in which evaluation of a new curcumin formulation by self-nano emulsifying drug delivery system on protection from pain and functional deficits associated with diabetic neuropathy was investigated [117]. The bioavailability of curcumin increased with encapsulation technique, and the study showed that curcumin reversed the functional, sensorimotor, and biochemical deficits by reducing neuroinflammation and enhancing antioxidant defense in diabetic neuropathy.

### 6.4. Effects on Diabetic Cardiomyopathy

Cardiovascular complications are known to be the main causes of disability and death in diabetic patients, among the different complications of diabetes. Diabetic cardiomyopathy is a structural and functional disorder involving deregulation of the metabolic pathway, left ventricular dysfunction, and disruption of myocardial cells [118]. Effective and novel therapeutic strategies for diabetes and diabetic cardiomyopathy have been searched by researchers. Accumulating clinical and preclinical studies have demonstrated that curcumin has positive effects on diabetic cardiomyopathy.

Panahi et al. [119] reported that curcumin has an antiatherosclerosis effect in diabetic patients. Curcumin intake (1,000 mg per day) for 3 months could contribute to decreasing serum non-high-density lipoprotein cholesterol (non-HDL-C) and lipoprotein A levels in patients with T2D.

In addition, Panahi et al. [120] proved that curcumin intake (1,000 mg per day) for 3 months increased adiponectin and decreased leptin level and leptin/adiponectin ratio (measurement of atherosclerosis) in patients which have T2D. In a preclinical trial, it was observed that the nanoencapsulation method has been increased solubility and bioactivity of the curcumin. Nanocapsulated curcumin alleviated diabetic cardiomyopathy with increasing hydrogen sulfide and Ca^2+^ levels and regulation of calcium-sensing receptor expression, endogenous cystathionine-*γ*-lyase (CSE), and calmodulin [109].

### 6.5. Effects on Diabetic Retinopathy

Diabetes can affect the eyes which can lead to vision problems or even blindness. Diabetic retinopathy damages the tiny blood vessels in the retina [121]. Curcumin can be used for the prevention of retina attenuation by enhancing the ultrastructure changes of the retina [122]. Another major complication of diabetes on the eyes is cataract which is distinguished by cloudiness or opacification of the crystalline eye lens.

Based on the preclinical studies, curcumin delays diabetic cataract in rats. Grama et al. [77] reported that the administration of 2 mg/day nanocurcumin delays the progression of diabetic cataracts in rats by the action of biochemical pathways of disease progression such as protein insolubilization, protein glycation, polyol pathway, crystallin distribution, and oxidative stress. Additionally, they reported that nanoencapsulation improved the oral bioavailability of curcumin.

## 7. Concluding Remarks

Diabetics are not able to metabolize well glucose due to defects in insulin secretion and/or action, and curcumin is capable to exert a therapeutic effect playing a critical role on *β*-cell functions. Several studies have been shown the high therapeutic properties of curcumin in hyperglycemia, diabetic wound healing, diabetic neuropathy, diabetic cardiomyopathy, and diabetic retinopathy and cataract. The therapeutic applications of curcumin to treat diseases have some limitations like poor solubility, low instability, low bioavailability, low penetration, rapid metabolization, and targeting efficacy. To overcome these limitations, researchers developed new delivery systems for curcumin. Studies of the new stabilizers and biopolymer-based drug delivery systems are needed to improve the possible cytotoxic effects, stability in different digestive juices, encapsulation efficiency, and drug release time. In addition, the design of new nanoformulations is necessary to expand the use of nanomedicine to the different routes of drug administration, such as intravenous, intranasal, and oral.

## Figures and Tables

**Table 1:** Some recent curcumin nanoformulations and beneficial effects on diabetic complications.

Type of nanoformulations^∗^	Method	Encapsulation efficiency (%)	Size (nm)	Beneficial effects	References
Curcumin nanoparticles loaded in Tween 60	Solvent evaporation nanoprecipitation	93	40-50	↓ Endothelial dysfunction	[103]
Curcumin-loaded liposomes coated with PEG	Film hydration	80	140	Anti-inflammatory	[54]
Curcumin-ZnO complex loaded with chitosan	Solvent evaporation followed by ion gelation	Not reported	43	↓ Blood glucose level maintain normal lipid profile ↑ Solubility of curcumin ↑ Antioxidant activity	[72]
Curcumin-loaded PLGA nanoparticles	Emulsion-diffusion-evaporation	66	237	Protective effect on inflammatory markers improve lipid metabolisms	[76]
Curcumin-loaded Pluronic nanomicelles	Nanoprecipitation	88-91	333	↑ *β*-Cell regeneration	[101]
Curcumin-loaded PLA-PEG nanoparticles	Emulsion-diffusion-evaporation	98	117	Protective effect on liver inflammation	[73]
Curcumin-loaded PLGA nanoparticles	Emulsion-diffusion-evaporation	56	282	Delaying diabetic cataracts	[77]
Curcumin-loaded nanoparticles coated with chitosan	Ion crosslinking	77	91	↑ Healing of diabetic wounds	[107]
Curcumin nanoparticles within QPAMAM (G3)	Emulsion solvent evaporation	Not reported	40	Preventing oxidant mediated diabetic cataract development	[108]
Curcumin-encapsulated PBLG-PEG-PBLG nanocapsules	Ring-opening polymerization	32	30	↓ Diabetic cardiomyopathy	[109]

Abbreviations and symbols: ↑: increase; ↓: decrease; PLA: polylactic acid; PEG: polyethylene glycol; PBLG: poly(gamma-benzyl l-glutamate); QPAMAM (G3): quaternary ammonium poly(amidoamine) dendrimer.

## Data Availability

The data supporting this review are from previously reported studies and datasets, which have been cited. The processed data are available from the corresponding author upon request.
